# Alpha-Gal Syndrome in a Military Member

**DOI:** 10.7759/cureus.72004

**Published:** 2024-10-21

**Authors:** Jackson L Howell, Brittanie Neaves, Christopher Coop

**Affiliations:** 1 Allergy and Immunology, 81st Medical Group Keesler Air Force Base, Biloxi, USA; 2 Allergy and Immunology, Keesler Medical Center, Biloxi, USA

**Keywords:** alpha-gal syndrome, amblyomma americanum, galactose-alpha-1, military healthcare, tick-borne infections

## Abstract

Alpha-gal syndrome is an acquired disease ranging from gastrointestinal discomfort to anaphylaxis, an acute, life-threatening allergic reaction. Susceptible individuals have high-risk vocations or hobbies that involve outdoor activities where tick populations are overabundant. Potential exposure increases if located in the southeastern United States where *Amblyomma americanum*, or the lone star tick, carryingα-gal glycoprotein is prevalent. We present a case of exposure to such a tick population and the subsequent diagnosis of alpha-gal syndrome. Our case brings to question both the complex management of this disease and treatment within the military sector.

## Introduction

Galactose-alpha-1, 3-galactose, or what is simply known as alpha-gal syndrome, is an immunological disease that is quickly becoming widespread throughout America. The culprit has proven to be *Amblyomma americanum*, also commonly known as the lone star tick, which is also notorious for the spread of bacteria such as *Rickettsia* and *Ehrlichia* and viruses including the heartland virus and the Bourbon virus. This parasite is located throughout the southeastern, midwestern, and eastern United States. However, the highest prevalence of alpha-gal-carrying tick populations resides in Arkansas (39%), Oklahoma (35%), and Missouri (29%) [1-3]. After one or two bites from this species, a person may develop a delayed allergic onset of symptoms ranging from three to eight hours after red meat consumption [4-7]. The aim of this article is to review a patient case of alpha-gal syndrome and its treatment.

## Case presentation

**Figure 1:**
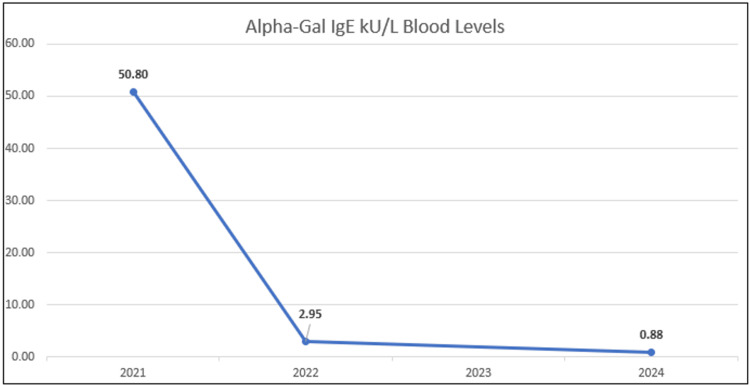
Alpha-galactose blood serology test Blood draws conducted 2021-2024.

A 42-year-old male, working in the US military, residing in Missouri in the summer of 2020, had a new onset of generalized urticaria (hives) located on his face, trunk, and limbs. On review of potential causes, he reports consumption of beef protein (i.e., steak) approximately four hours before the onset of symptoms. His history did not suggest other allergic etiologies, such as a known history of food allergy, drug allergy, an insect sting, or latex allergy. He is an avid hunter within Missouri, where the Lonestar tick thrives, and he reports a tick bite earlier that spring while on a hunting trip in the local outdoors. On further evaluation, skin testing was conducted and revealed an allergic sensitivity to beef extract and serum testing revealed an alpha-galactose IgE serology level of 50.80 kU/L (positive is ≥ 0.35 kU/L) (Figure 1).

The patient was instructed to avoid mammalian meats, which include beef and pork, and carry an epinephrine auto-injector in case of anaphylaxis. A beef challenge was not offered to the patient at this time.

The patient had a history of seasonal spring and fall allergic rhinoconjunctivitis symptoms to pollen and grass for several years. His symptoms included rhinorrhea, nasal congestion or dripping, and itchy watery eyes. The patient took a daily tablet of 180 mg of fexofenadine as needed during environmental exposures.

In April 2024, after moving to Biloxi, Mississippi, the patient was referred to the Keesler Medical Center Allergy Clinic to establish care with a new allergist for his alpha-gal allergy. After avoiding mammalian meats for three years, he wished to retest for the allergy in order to reincorporate bacon and steak into his diet. The patient also denied having tick bites during this period. Repeat testing resulted in an alpha-gal IgE level of 0.88 kU/L, a beef IgE of 0.14 kU/L, and a pork IgE of 0.15 kU/L. The patient underwent an oral food challenge with bacon and steak on separate visits and passed both challenges without immediate or delayed symptoms of urticaria, angioedema, or other anaphylaxis symptoms. He was observed for six hours during the oral food challenges.

## Discussion

Alpha-gal allergy is an IgE-mediated disease with specific IgE directed to the carbohydrate moiety galactose-alpha-1 and 3-galactose (alpha-gal) located in mammalian meats. Patients sensitized to alpha-gal typically present with delayed symptoms of urticaria, angioedema, and anaphylaxis, especially with ingestion of beef, pork, or lamb. Reactions may also occur when patients are exposed to mammalian-derived gelatin ingredients in foods, candies, and organ meats (i.e., liver, kidney) [1-4].

**Figure 2:**
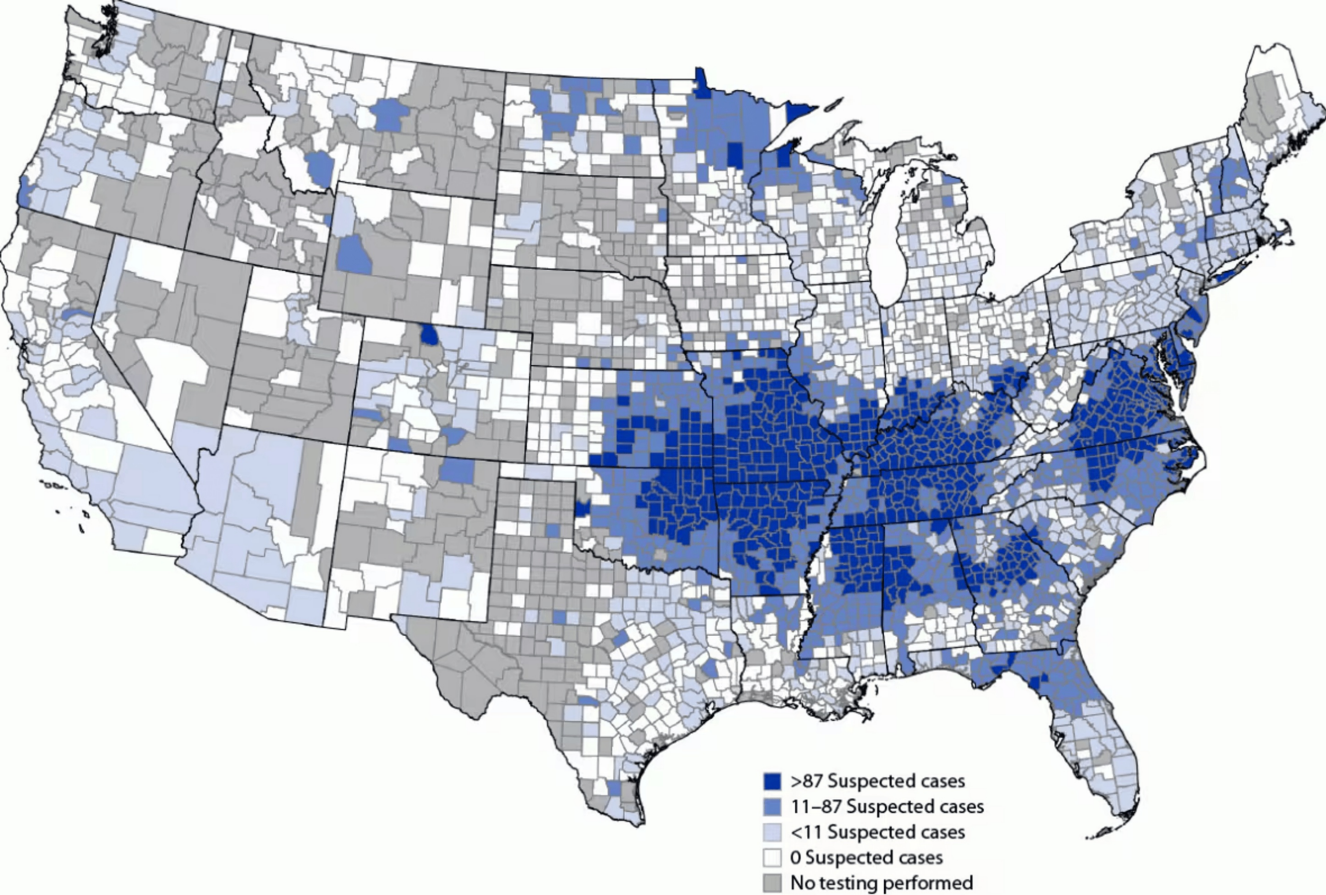
Geographic distribution of potential alpha-gal symptomatic incidence (Jan 2017-Dec 2022) Copyright/license: This figure has been adapted from Thompson et al. [5].

Alpha-gal allergy is most common in adults, but children may also be affected. In addition to urticaria, angioedema, and anaphylaxis, patients may have isolated gastrointestinal symptoms, such as abdominal cramping, vomiting, and diarrhea. Presyncope and syncope have been reported in some patients. The incidence of alpha-gal allergy continues to increase in the US and has also been identified in Europe, Asia, South Africa, and Australia [3,4]. The lone star tick responsible for sensitizing individuals to alpha-gal, populates predominantly in the midwest and southeast regions of the United States and peaks in the spring months of March through May. Please see Figure 2 for the geographic distribution of alpha-gal incidence.

Patients with alpha-gal tend to exhibit delayed symptoms, usually occurring three to eight hours after ingestion of the culprit food allergen. The delay may be related to the binding of the alpha-gal allergen to lipids in the gut, which are absorbed at a slower rate when compared to proteins [7]. Patients sensitized to alpha-gal may also react when exposed to the monoclonal antibody cetuximab, gelatin administered in vaccines, intravenous colloids, heparin, and bovine or porcine heart valves [8-11]. Alpha-gal allergy appears to resolve over time especially when patients are able to avoid tick bites [12].

The diagnosis of alpha-gal allergy can be confirmed by serum IgE testing. Avoidance of beef, pork, and other mammalian meats is recommended for symptomatic patients. Additionally, patients should carry an epinephrine autoinjector for anaphylaxis treatment in the unfortunate event of accidental ingestion of the culprit allergen. Our patient successfully avoided beef and pork and tick bites for two years. After retesting his alpha-gal IgE level, he was able to pass an oral food challenge to his favorite foods, bacon and steak. His quality of life has drastically improved since reincorporating these foods into his diet, and he no longer needs to carry an epinephrine autoinjector. Patients with an alpha-gal allergy may participate in military deployments if their symptoms are not severe. Most military members with alpha-gal allergy remain in the military and are able to continue their duties and responsibilities.

## Conclusions

We present an interesting case of a patient who experienced an allergic reaction following beef consumption months after suffering from a tick bite on a hunting trip in a lone star tick endemic area. Initially, his alpha-gal IgE serology level was exceedingly elevated; however, after three years of tick, beef, and pork-avoidance measures, his IgE serology level reduced significantly, allowing him to undergo an oral food challenge and subsequent normalization of his previous diet.
